# The Origin of Cultivation and Proto-Weeds, Long Before Neolithic Farming

**DOI:** 10.1371/journal.pone.0131422

**Published:** 2015-07-22

**Authors:** Ainit Snir, Dani Nadel, Iris Groman-Yaroslavski, Yoel Melamed, Marcelo Sternberg, Ofer Bar-Yosef, Ehud Weiss

**Affiliations:** 1 The Martin (Szusz) Department of Land of Israel Studies and Archaeology, Institute of Archaeology, Bar Ilan University, Ramat-Gan, Israel; 2 The Zinman Institute of Archaeology, Haifa University, Haifa, Israel; 3 The Mina and Everard Goodman Faculty of Life Sciences, Bar-Ilan University, Ramat-Gan, Israel; 4 Department of Molecular Biology and Ecology of Plants, The George S. Wise Faculty of Life Sciences, Tel Aviv University, Tel Aviv, Israel; 5 Department of Anthropology, Harvard University, Cambridge, Massachusetts, United States of America; Kunming Institute of Botany, CHINA

## Abstract

Weeds are currently present in a wide range of ecosystems worldwide. Although the beginning of their evolution is largely unknown, researchers assumed that they developed in tandem with cultivation since the appearance of agricultural habitats some 12,000 years ago. These rapidly-evolving plants invaded the human disturbed areas and thrived in the new habitat. Here we present unprecedented new findings of the presence of “proto-weeds” and small-scale trial cultivation in Ohalo II, a 23,000-year-old hunter-gatherers' sedentary camp on the shore of the Sea of Galilee, Israel. We examined the plant remains retrieved from the site (ca. 150,000 specimens), placing particular emphasis on the search for evidence of plant cultivation by Ohalo II people and the presence of weed species. The archaeobotanically-rich plant assemblage demonstrates extensive human gathering of over 140 plant species and food preparation by grinding wild wheat and barley. Among these, we identified 13 well-known current weeds mixed with numerous seeds of wild emmer, barley, and oat. This collection provides the earliest evidence of a human-disturbed environment—at least 11 millennia before the onset of agriculture—that provided the conditions for the development of "proto-weeds", a prerequisite for weed evolution. Finally, we suggest that their presence indicates the earliest, small-scale attempt to cultivate wild cereals seen in the archaeological record.

## Introduction

The search for concrete evidence for the first appearance of weeds some 12,000 years ago, when intentional systematic cultivation was initiated in the Levant, needs to rely on the prehistoric archaeobotanical plant assemblages [1–3]. The archaeological record demonstrates that pre-Neolithic human societies were hunter-gatherers for millennia when a radical development took place throughout Eurasia at the onset of the Holocene, 11,700/500 cal BP. Over the course of the next several millennia, foraging societies across the Fertile Crescent began cultivating as well as herding, tending goats, sheep, pigs, and cattle [2,4]. Eventually the development of the initial agricultural system saw the ‘Domestication Syndrome’ of both plants and animals. The establishment of long-term, agricultural-based permanent villages resulted in a population increase which later caused human expansions westward and eastward [5].

Research of the Paleolithic period has already demonstrated that humans caused significant modifications to their immediate environments long before the Neolithic revolution. This process that has intrigued scholars since the early 1950’s [6], and is currently referred as ‘niche construction’ [7–10]. Humans set fire to vegetation, hunted and trapped preferred species of mammals, birds, reptiles and fish, cut down trees for buildings and producing numerous objects, and created dump areas in and around their camps. Later, with the onset of successful intentional cultivation, hunter-gatherers cleared fields near their habitations for planting. The intensive disturbance of these environments led to the proliferation of synanthropic plants. These plant species, both annuals and perennials, exhibit functional and adaptive traits that enable them to withstand the disturbed habitats and increase their biological fitness in natural plant communities altered by natural or anthropogenic forces [3]. Owing to their rapid water uptake (particularly in water-limited habitats), high growth rates, dispersal capabilities, and ability to thrive in areas with altered soil nutrient resources, synanthropic species (later termed weeds) frequently invade newly formed habitats [11,12]. They were able to rapidly established extensive populations, especially with the expansion of farming by invading cultivated fields and causing reduced crop yields.

Although modern agronomists and archaeologists generally refer to weeds as plants present in field crops, this term needs to be carefully defined as its use is manifold in both research domains. Here, weeds are defined as plants that disrupt or alter the functioning and composition of natural ecosystems and human-altered environments. In most cases in the Near East and Europe, they negatively impact human activities and as such are undesirable. “Proto-weeds” are defined as the first wild plants that entered and thrived in early human-affected habitats, which subsequently led to the evolution of weeds [13].

Because weeds thrive in cultivated fields and disturbed soils [14], a *significant* presence of weeds in archaeobotanical assemblages retrieved from Neolithic sites and settlements of later age is widely considered an indicator of systematic cultivation [15–20]. Generally, weeds are useful ecological markers *only* if they are identified to the species level. Undoubtedly, in genera which include several species, each species could have an entirely different ecological signature and could not serve as a proxy for agricultural activity [21]. The unique anaerobic conditions that prevailed at Ohalo II enabled the high level of preservation of the samples, allowing the identification to the species level.

Until now, the geographic origin of some current Southwest Asian weeds was unknown. In this paper we present archaeobotanical evidence indicating that some of these species were initially present in human-affected environments as local wild plants during the Terminal Pleistocene. Later, with the establishment of systematic farming, they evolved into weeds or functioned as weeds without further evolution. We term the first stage of this human-plant interaction as “the *proto-weed* stage”.

## Materials and Methods

**Fig 1:**
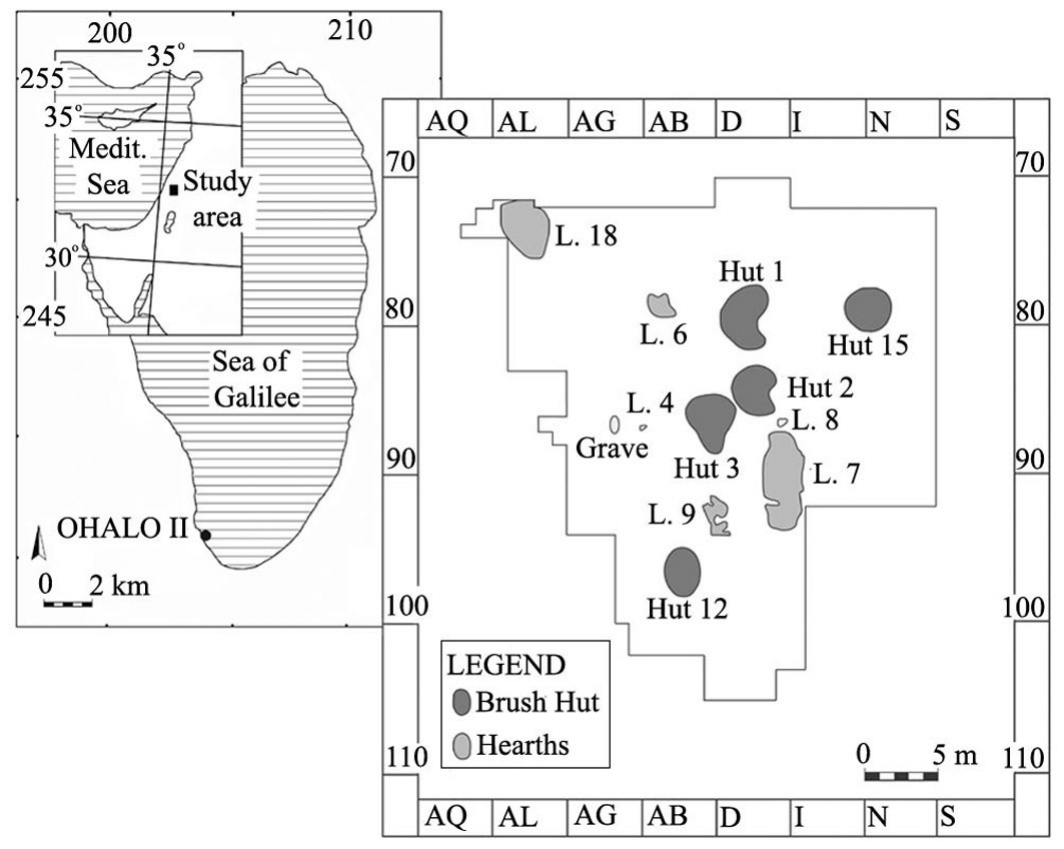
Location map of Ohalo II and central area of excavation at the site.

Ohalo II is located on the southwestern shore of the Sea of Galilee (Lake Kinneret), Israel. The site was inhabited during the Last Glacial Maximum (LGM) at ~23,000 years ago and then inundated [22]. It was discovered in 1989 when the lake water level dropped drastically following several years of drought and intensive water pumping. The submerged site was excavated during six seasons from 1989–1991 and 1998–2001 (Excavation permits: 1989: L. 1634; 1990: L. 1724; 1991: 93/91; 1998: 160/98; 1999: G-61/99; 2000: G-20/2000; 2001: G-73/2001, issued by the Israel Antiquities Authority), and the uncovered huts were exceptionally well-preserved. The remains of six brush huts were identified during excavations, in addition to several hearths (Fig 1). Four of the brush huts were fully excavated and two were only sampled. All of the huts had a bowl-like cross section, with the floor dug below ground level. Four huts were oval in shape, while two were kidney-shaped.

The plant species used for hut construction were identified in Brush Hut 1; it was built with thick branches of *Tamarix* (tamarisk), *Salix* (willow) and *Quercus ithaburensis* (Mt. Tabor oak), covered by smaller branches of other woody species such as *Atriplex/Seidlitzia* (orach/seidlitzia) and *Prosopis* (mesquite), as well as by leaves and grasses. In the carefully floated and hand-picked deposits found in these huts, a particularly rich plant assemblage of *ca*. 150,000 seeds and fruits was collected. Various studies of this assemblage have already been published [22–26]. In addition, a wide variety of flint and ground stone tools, faunal remains (fish, mammals, birds, rodents, reptiles, and mollusks), beads, bone, and wood objects were found on the floors of the huts. One grave of an adult male was found near one of the huts [27–40].

## Results and Discussion

### The site of Ohalo II and its finds

The outstanding preservation of the various assemblages has been discussed in several publications that suggest the rise of the lake level occurred immediately after the abandonment of this camp and sealed it for thousands of years [27,33,41,42]. The plant material was initially preserved by charring and the sedimentation of silts sealing them in the low-oxygen conditions under the lake water, an ideal setting for the preservation of the organic material [24,43].

More than one-third of this rich plant assemblage of seeds and fruits belongs to the grass family (Poaceae), including wild emmer wheat, wild barley, and wild oat (*Triticum dicoccoides*, *Hordeum spontaneum*, *Avena barbata/sterilis*). Unequivocal evidence that these grains were processed for consumption is provided by (**i**) a grinding slab set on the floor of Brush Hut 1 from which wild cereal starch granules were extracted, and (**ii**) by the patterned distribution of these species’ seeds found around the grinding slab [23,24,44].

Ohalo II functioned as a year-round settlement, as indicated by the remains of migratory birds known to visit the region in different seasons, and the timing of ripening of the identified plant remains [23,38] (S1 Fig). This conclusion is supported by the earliest occurrence of commensal species at the site, such as the house mouse (*Mus musculus*) and the rat (*Rattus rattus*)[33].

The plants and faunal remains demonstrate that Ohalo II inhabitants practiced a broad spectrum of exploitation of annual plants (Table 1) and birds [45]. Some of the plants are the progenitors of domesticated crop species such as emmer wheat, barley, pea, lentil, almond, fig, grape, and olive. Thus, about 11,000 years before what had been generally accepted as the onset of agriculture, people’s diets relied heavily on the same variety of plants that would eventually become domesticated.

**Table 1:** Main edible annual plant species from Ohalo II.

Family	Species	Identified seeds (n)
Poaceae (cereals)		9,703
	*Avena barbata*	
	*Avena sterilis*	
	*Hordeum spontaneum*	
	*Triticum dicoccoides*	
Poaceae (grasses)		27,994
	*Aegilops geniculata/peregrina*	
	*Bromus pseudobrachystachys*	
	*Hordeum marinum/hystrix*	
	*Piptatherum holciforme*	
	*Piptatherum blancheanum*	
	*Puccinellia convoluta*	
Fabaceae		70
	*Lathyrus* sp.	
	*Lens* sp.	
	*Pisum sativum* ssp. *humile*	
	*Vicia palaestina*	
**Total**		**37,767**

### The proto-weeds

Among the *ca*. 150,000 identified charred seeds and fruits, 15,726 (10.5%) of these seeds belong to 13 current weed species (Table 2). Their high frequency reflects their common presence as proto-weeds within the immediate environment of the site. Almost all of the studied proto-weed seeds (93.2%) belong to two important species of current weeds in crop fields: corn cleavers (*Galium tricornutum*), and ten grains of darnel (cf. *Lolium temulentum*) (S2 Fig). Until now, the original habitat of these plants was unknown as they are rare outside segetal environments in the Levant [14]. Ohalo II therefore provides the oldest and clearest known indication of their origin, as well as the time of their entrance into human-made niches [7–9,46]. Also, it is possible that we couldn’t identify the darnel grain in 100% (hence the ca.) because these grains are morphologically closer to its unknown wild-type than the current weed-type. Four weed species found at the site, *Chenopodium album*, *Malva parviflora*, *Notobasis syriaca*, and *Silybum marianum* are common current weeds of the region that are typically found in disturbed areas or dump areas, though some of their parts are edible[14,47–52]. Due to the presence of edible plants in this last group, they may have been gathered for consumption in the wild or from a local dump area where they grew.

**Table 2:** Proto-weed species from Ohalo II: current weeds in cultivated fields.

Species (organ)	Family	Identified seeds (n)
*Adonis dentata/microcarpa* ^a^ (seed)	Ranunculaceae	120
*Fumaria densiflora*/*parviflora* ^a^ (seed)	Fumariaceae	46
*Fumaria macrocarpa* (seed)	Fumariaceae	88
*Galium tricornutum* (fruitlet)	Rubiaceae	11,845
*Lolium rigidum/multiflorum* ^a^ (grain)	Poaceae	7
cf. *Lolium temulentum* (grain)	Poaceae	10
*Melilotus indicus* (seed)	Fabaceae	285
*Neslia apiculata* (fruitlet)	Brassicaceae	132
**Edible weeds**		
*Chenopodium album* (seed)	Chenopodiaceae	613
*Malva parviflora* (fruitlet)	Malvaceae	1,030
*Malva parviflora*/*aegyptiaca* ^a^ (fruitlet)	Malvaceae	1,200
*Notobasis syriaca* (seed)	Asteraceae	24
*Silybum marianum* (seed)	Asteraceae	326
**Total weeds**		**15,726**

^a^The taxa identified as two closely related species in this table are both weeds. Some of these plants are edible.

The presence of such a wide variety and huge quantity of proto-weeds, particularly *Galium tricornutum*, might indicate that these species were growing together with the cereals collected for consumption (wild barley, wild wheat, and wild oat). Since these cereals and weeds currently grow within the anthropogenic niche of wild and cultivated fields in the Jordan Valley, and in light of their common presence in Ohalo II (Table 2), we should consider two alternative hypotheses. First, that they were collected in the wild, and second, that they grew in trial plots as the locals engaged in small-scale, elementary cereal cultivation.

In addition, the findings of weeds such as *Ch*. *album*, *M*. *parviflora*, *N*. *syriaca*, and *S*. *marianum* (Table 2) provide the earliest botanical indication of a disturbed environment in the Levant as a dump area where nitrophilic proto-weeds proliferated [8]. This altered environment favored the establishment and growth of these species that flourished in the disturbed environment of the immediate surroundings of the Ohalo II’s permanent camp.

**Fig 2:**
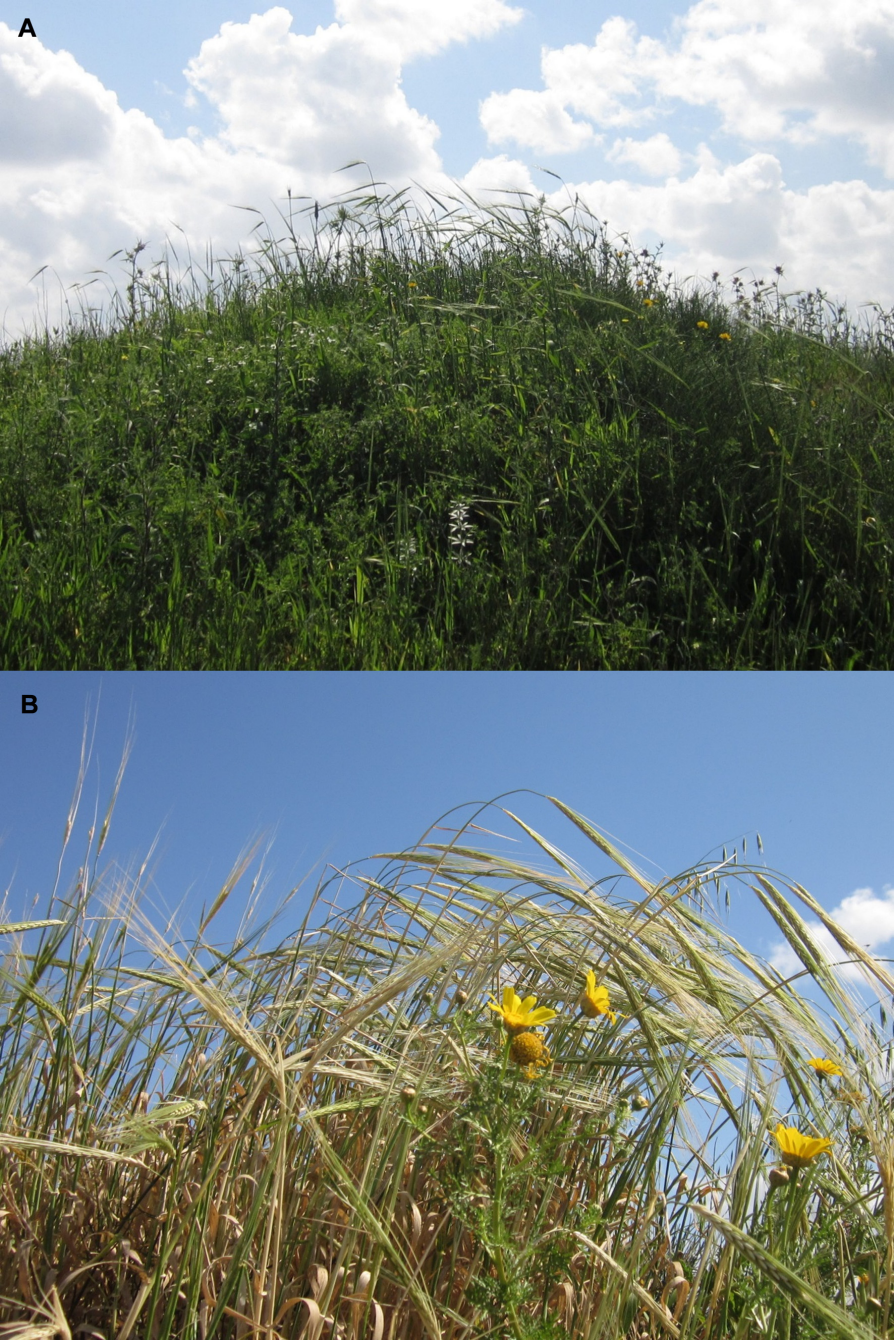
Wild barley (*Hordeum spontaneum*). (A) Wild barley field in Yakum Park (32° 14′ 50.28″ N, 34° 50′ 33″ E. March 18, 2013). It grows here with other species such as *Galium aparine*, *Chrysanthemum coronarium*, *Notobasis syriaca*, and *Anthemis* sp. (B) Same field, showing wild barley at three ripening stages – green, green-yellow and yellow.

**Fig 3:**
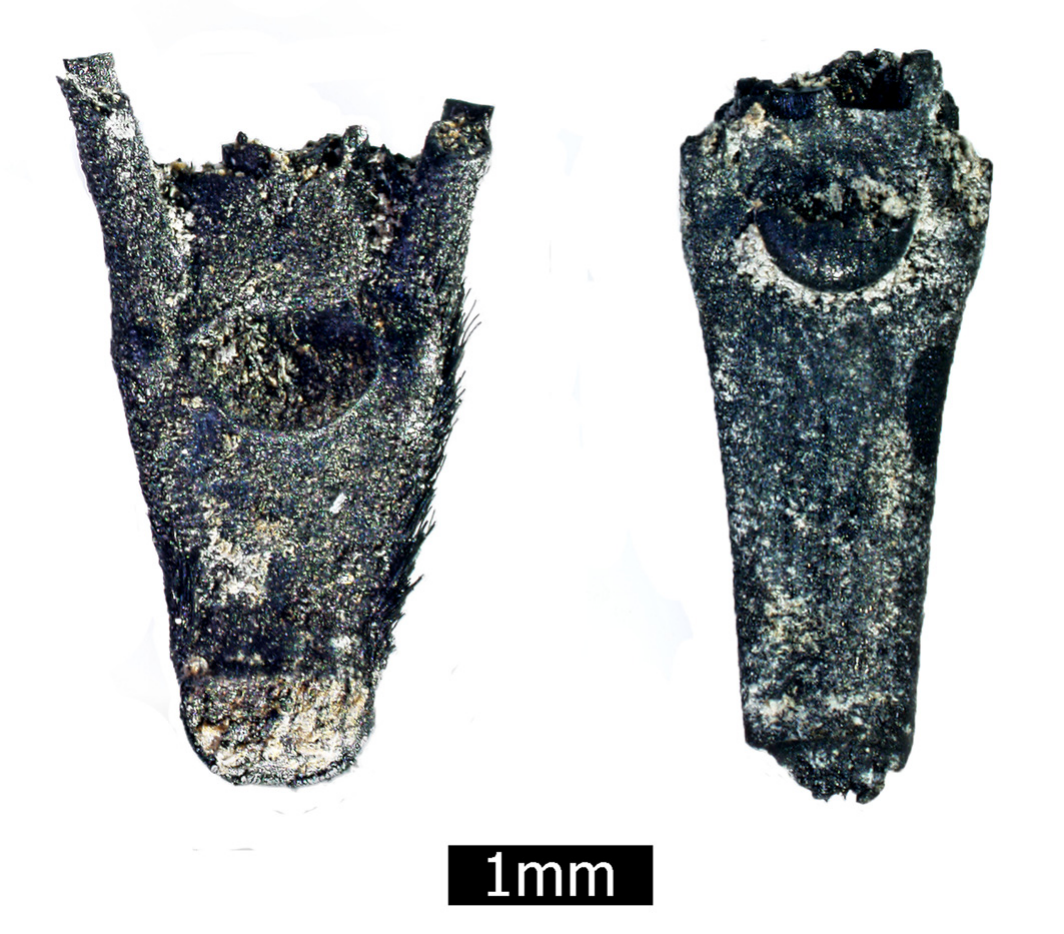
Wild-type (left) and domestic-type (right) scars in rachises of wild barley (*Hordeum spontaneum*) from Ohalo II.

Here we note that domesticated cereals are usually identified by the type of disarticulation scars in their rachises; smooth disarticulation scars are considered as the main diagnostic element for the identification of the wild forms, while rough disarticulation scars indicate the domestic form [2]. Presence of >10% domestic-type in a given archaeobotanical assemblage is regarded as an indication for domestication [53,54]. At Ohalo II, 320 wild barley rachises were found, of which 36% show domestic-type scars (Fig 3), alongside 148 wild wheat rachises, 25% of which are domestic-type scars. Importantly, the relative width of the rachises indicate that most of the domestic-type scars do not derive from the lower part of the ear [54]. Traditionally, similar finds would be regarded as domesticated. However, we do not claim such status. First, ears of wild cereals mature gradually, with the upper spikelet ripening and shedding to the ground [55]. Second, ear ripening in wild stands is so irregular that at any given time only some of the ears are yellow (fully ripe), while others are green (unripe), or in the intermediate stage of green-yellow [56] (Fig 2). However, field studies conducted in wild barley populations across Israel showed that harvested green or green-yellow ears tend to disarticulate and show the same wild-type clean scars when allowed to dry, rather than domestic-type rough scars [54,57]. This high percentage of domestic-type scars in the Ohalo II assemblage is significant, therefore, to our reconstruction of the site inhabitants’ practices.

**Fig 4:**
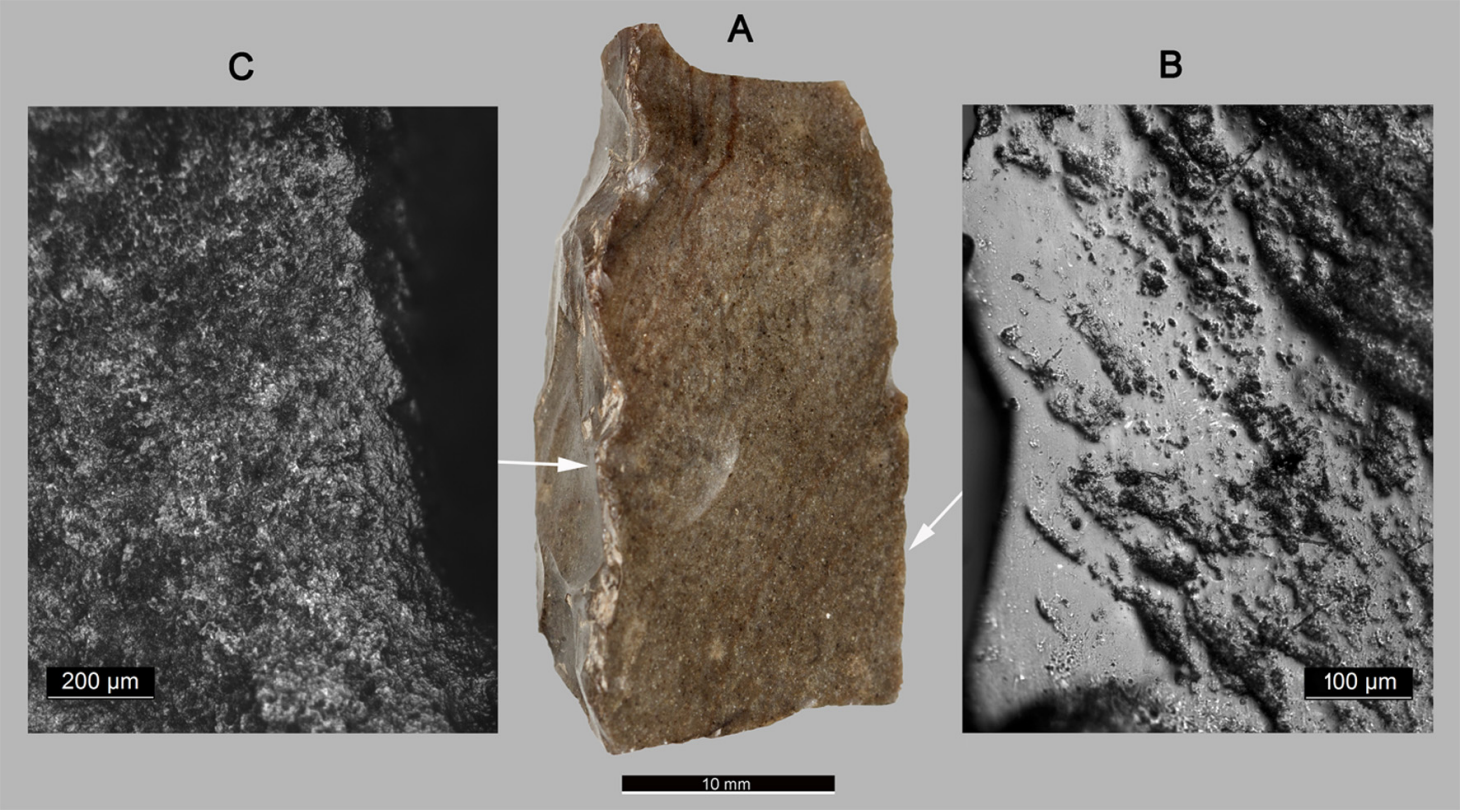
The sickle blade from Ohalo II. (A) Macrograph of the sickle blade. (B) Micrograph showing the use-wear polish produced by cereal harvesting, observed along the sharp edge of the blade (original magnification 200x). (C) Micrograph showing hafting wear including streaks of polish associated with rounding observed along the opposite edge (original magnification 100x).

Harvesting green-yellow cereal fields at Ohalo II is supported by the study of glossed flint blades found at the site. Five implements are currently being studied, one of which is presented here (Fig 4). According to experimental results we conducted on wild cereal stands, it is concluded that the use-wear polish observed on the sharp edges was produced by cutting green-yellow cereal stems. The characteristics of the microscopic traces resemble those found on Natufian sickle blades, characterized by a relatively thin band of bright and smooth polish, indicating a short harvesting action [58–61]. The glossed blade (Fig 4) also bears hafting traces on the opposite edge, including streaks of polish and rounding of elevated surfaces typical of a flint implement hafted longitudinally, indicating that such a blade was a part of a composite tool, perhaps one of several blades in a long handle. Although sickle blades are very rare in pre-Natufian sites, they provide clear evidence for harvesting techniques using pre-planned composite tools.

In other words, these sickle blades indicate that the Ohalo II people harvested cereals in the same manner previously known only from the Natufian period, some 10 millennia later.

## Conclusions

We suggest that the Ohalo II archaeobotanical remains indicate that the locals practiced small-scale cultivation, with no evidence for its continuation in the following period. Similar failed trials with new techniques are known from the history of technology but the ideas remain within the generally same population [62]. There are several lines of evidence supporting this suggestion: (i) The large numbers of edible grasses, wild wheat, wild barley and wild oat; (ii) The large numbers of proto-weeds; (iii) The common presence of domestic-type disarticulation scars, far beyond the normal representation in wild populations, and (iv) The presence of the earliest sickle blades, indicating planned cereal harvesting. Indeed, it is important to note that we do not claim a domesticated status for the Ohalo II wheat and barley. We assume that such trial cultivation could be the reason for the significant representation of domestic-type rachises. In turn, the domestic-type rachis is probably an indication of some evolutionary change, similar to the process that took place some ten millennia or so later, when wheat and barley became fully domesticated. However, these changes likely disappeared after the short Ohalo II cultivation endeavor, although archaeobotanical data (and sickle blades) are still lacking from the immediately succeeding sites

It was already suggested [2] that the appearance of non-shattering mutants in cereal crops was a fast process. Genetic considerations indicate that such a shift could have been accomplished in the course of a few dozen generations of selection [63–67]. If so, the Ohalo II assemblage containing a high percentage of domestic-type rachises, might be the earliest indication for fast domestication rate [68].

In conclusion, our findings represent the earliest indications for the presence of proto-weeds in a site predating the Neolithic plant domestication by some 11,000 years. This study shows for the first time that proto-weeds grew in the vicinity of human camps and most probably also in small scale, cultivated plots. Further discoveries of well-preserved archaeobotanical assemblages from the period between Ohalo II and the first Neolithic sites in the region will provide the missing evidence of trials and failures in early agriculture.

## Supporting Information

S1 Fig Seasonal availability of plants and birds found at Ohalo II, based on 68 species of birds [38]] and 101 species of plants. These two types of food resources compensate for each other–birds are more available during autumn/winter while plants prevail in spring/summer. The circled numbers indicate the number of migratory bird species that currently visit the area only once a year. These were eaten from December to June at Ohalo II, and therefore serve as a good indicator of season of occupation of the site. These and other faunal remains point to year–round occupation of Ohalo II.
(DOCX)

S2 Fig Darnel (*Lolium temulentum*). (A) Modern darnel and (B) archaeological darnel from Ohalo II.
(DOCX)

## References

[1] Doebley JF, Gaut BS, Smith BD (2006) The Molecular Genetics of Crop Domestication. Cell 127: 1309–1321. 17190597 10.1016/j.cell.2006.12.006

[2] Zohary D, Hopf M, Weiss E (2012) Domestication of Plants in the Old World: The origin and spread of domesticated plants in Southwest Asia, Europe, and the Mediterranean Basin Oxford, UK: Oxford University Press.

[3] Alpert P, Bone E, Holzapfel C (2000) Invasiveness, invasibility and the role of environmental stress in the spread of non-native plants. Perspectives in Plant Ecology, Evolution and Systematics 3: 52–66.

[4] Zeder MA (2011) The Origins of Agriculture in the Near East. Current Anthropology 52: S221–S235.

[5] Bouquet-Appel J-P (2011) The agricultural demographic transition during and after the agricultural inventions. Curr Anthropol 52: S497–S449S.

[6] Thomas WL, editor (1956) Man's role in changing the face of the earth Chicago: University of Chicage Press.

[7] Smith BD (2007) Niche construction and the behavioral context of plant and animal domestication. Evolutionary Anthropology: Issues, News, and Reviews 16: 188–199.

[8] Smith BD (2011) General patterns of niche construction and the management of ‘wild’ plant and animal resources by small-scale pre-industrial societies. Philosophical Transactions of the Royal Society B Biological Sciences 366: 836–848. 10.1098/rstb.2010.0253 PMC3048989 21320898

[9] Smith BD, Zeder MA (2013) The onset of the Anthropocene. Anthropocene 4: 8–13.

[10] Jones CG, Lawton GH, Shachak M (1994) Organisms as ecosystem engineers. Oikos 69: 373–386.

[11] Daehler CC (2003) Performance comparisons of co-occurring native and alien invasive plants: implications for conservation and restoration. Annu Rev Ecol Evol Syst 34: 183–211.

[12] Davis MA, Grime JP, Thompson K (2000) Fluctuating resources in plant communities: a general theory of invasibility. Journal of Ecology 88: 528–534.

[13] Delcourt HR (1987) The impact of prehistoric agriculture and land occupation on natural vegetation. Trends in Ecology & Evolution 2: 39–44. 21227814 10.1016/0169-5347(87)90097-8

[14] Zohary M (1950) The segetal plant communities of Palestine. Vegetatio 2: 387–411.

[15] Willcox G (2012) Searching for the origins of arable weeds in the Near East. Vegetation History and Archaeobotany 21: 163–167.

[16] Harlan JR, de Wet JMJ (1965) Some thoughts about weeds. Economic Botany 19: 16–24.

[17] Hillman GC, Hedges R, Moore AMT, Colledge SM, Pettitt P (2001) New evidence of Lateglacial cereal cultivation at Abu Hureyra on the Euphrates. Holocene 11: 383–393.

[18] Jones G (2002) Weed ecology as a method for the archaeobotanical recognition of crop husbandry practices. Acta Palaeobotanica 42: 185–193.

[19] Chernoff MC, Paley SM (1998) Dynamics of Cereal Production at Tell el Ifshar during the Middle Bronze Age. Journal of Field Archaeology 25: 397–416.

[20] Chernoff MC (1988) The Archaeobotanical Material from Tel el Ifshar. Waltham, Massachusetts: Brandeis University.

[21] Hartmann-Shenkman A, Kislev M, Galili E, Melamed Y, Weiss E (2015) Invading a new niche: obligatory weeds at Neolithic Atlit-Yam, Israel. Vegetation History and Archaeobotany 24: 9–18.

[22] Nadel D, Weiss E, Simchoni O, Tsatskin A, Danin A, et al (2004) Stone Age hut in Israel yields world's oldest evidence of bedding. Proceedings of the National Academy of Sciences of the United States of America 101: 6821–6826. 15090648 10.1073/pnas.0308557101 PMC404215

[23] Piperno DR, Weiss E, Holst I, Nadel D (2004) Processing of wild cereal grains in the Upper Palaeolithic revealed by starch grain analysis. Nature 430: 670–673. 15295598 10.1038/nature02734

[24] Nadel D, Piperno DR, Holst I, Snir A, Weiss E (2012) New evidence for the processing of wild cereal grains at Ohalo II, a 23 000-yearold campsite on the shore of the Sea of Galilee, Israel. Antiquity 86: 990–1003.

[25] Weiss E, Kislev ME, Simchoni O, Nadel D (2005) Small-grained wild grasses as staple food at the 23,000 year old site of Ohalo II, Israel. Economic Botany: S125-S134.

[26] Snir A, Nadel D, Weiss E (2015) Plant-food preparation on two consecutive floors at Upper Paleolithic Ohalo II, Israel. Journal of Archaeological Science 53: 61–71.

[27] Nadel D (2002) Ohalo II—A 23,000-Year-Old Fisher-Hunter-Gatherers' Camp on the Shore of the Sea of Galilee. Haifa: Hecht Museum, University of Haifa, Catalogue No. 20.

[28] Rabinovich R (2002) The mammal bones: environment, food and tools In: Nadel D, editor. Ohalo II—A 23,000-Year-Old Fisher-Hunter-Gatherers' Camp on the Shore of the Sea of Galilee. Haifa: Hecht Museum, University of Haifa, Catalogue No. 20. pp. *24–*27.

[29] Simmons T (2002) The birds from Ohalo II In: Nadel D, editor. Ohalo II—A 23,000-Year-Old Fisher-Hunter-Gatherers' Camp on the Shore of the Sea of Galilee. Haifa: Hecht Museum, University of Haifa, Catalogue No. 20. pp. *32–*36.

[30] Belmaker M (2002) The small mammals from Ohalo II and the environment, "Our mice that mar the land" (Samuel 1, VI:5) In: Nadel D, editor. Ohalo II—A 23,000-Year-Old Fisher-Hunter-Gatherers' Camp on the Shore of the Sea of Galilee. Haifa: Hecht Museum, University of Haifa, Catalogue No. 20. pp. *37–*38.

[31] Zohar I (2002) Fish and fishing at Ohalo II In: Nadel D, editor. Ohalo II—A 23,000-Year-Old Fisher-Hunter-Gatherers' Camp on the Shore of the Sea of Galilee. Haifa: Hecht Museum, University of Haifa, Catalogue No. 20. pp. *28–*31.

[32] Nadel D, Danin A, Werker E, Schick T, Kislev ME, Stewart K (1994) 19,000-year-old twisted fibers from Ohalo II. Current Anthropology 35: 451–458.

[33] Nadel D, Tsatskin A, Belmaker M, Boaretto E, Kislev M, Mienis H, et al (2004) On the shore of a fluctuating lake: Environmental evidence from Ohalo II (19,500 B.P.). Israel Journal of Earth Sciences 53: 207.

[34] Hershkovitz I, Edelson G, Spiers M, Arensburg B, Nadel D, Levi B (1993) Ohalo II man—unusual findings in the anterior rib cage and shoulder girdle of a 19000-year-old specimen. International Journal of Osteoarchaeology 3: 177–188.

[35] Rabinovich R, Nadel D (1995) Bone tools from Ohalo II—a morphological and functional study. Mitekufat Haeven, Journal of Israel Prehistoric Society 26: 32–63.

[36] Hershkovits I, Spiers MS, Frayer D, Nadel D, Wish-Baratz S, Arensburg B (1995) Ohalo II H2: A 19,000-year-old skeleton from a water-logged site at the Sea of Galilee, Israel. American Journal of Physical Anthropology 96: 215–234. 7785722 10.1002/ajpa.1330960302

[37] Kislev ME, Nadel D, Carmi I (1992) Epipalaeolithic (19,000 BP) cereal and fruit diet at Ohalo II, Sea of Galilee, Israel. Review of Palaeobotany and Palynology 73: 161–166.

[38] Simmons T, Nadel D (1998) The avifauna of the early Epipalaeolithic site of Ohalo II (19 400 years BP), Israel: species diversity, habitat and seasonality. International Journal of Osteoarchaeology 8: 79–96.

[39] Zohar I, Belmaker M, Nadel D, Gafny S, Goren M, Hershkovits I, et al (2008) The living and the dead: How do taphonomic processes modify relative abundance and skeletal completeness of freshwater fish? Palaeogeography, Palaeoclimatology, Palaeoecology 258: 292–316.

[40] Nadel D, Hershkovitz I (1991) New subsistence data and human remains from the earliest Levantine Epipalaeolithic. Current Anthropology 32: 631–635.

[41] Nadel D (2002) Indoor / outdoor flint knapping and minute debitage remains: the evidence from the Ohalo II submerged camp (19.5 ky, Jordan Valley). Lithic Technology 26: 118–137.

[42] Nadel D, Carmi I, Segal D (1995) Radiocarbon dating of Ohalo II: archaeological and methodological implications. Journal of Archaeological Science 22: 811–822.

[43] Weiss E (2002) Issues in reconstruction the human economy and society of the Epipalaeolithic site Ohalo II inhabitants by the macrofossil botanical remains [Ph.D.]. Ramat-Gan: Bar-Ilan University.

[44] Weiss E, Kislev ME, Simchoni O, Nadel D, Tschauner H (2008) Plant-food preparation area on an Upper Paleolithic brush hut floor at Ohalo II, Israel. Journal of Archaeological Science 35: 2400–2414.

[45] Weiss E, Wetterstrom W, Nadel D, Bar-Yosef O (2004) The broad spectrum revisited: Evidence from plant remains. Proceedings of the National Academy of Sciences of the United States of America 101: 9551–9555. 15210984 10.1073/pnas.0402362101 PMC470712

[46] Smith BD (2007) The Ultimate Ecosystem Engineers. Science 315: 1797–1798. 17395815 10.1126/science.1137740

[47] Feinbrun-Dothan N, Danin A (1991) Analytical flora of Eretz-Israel. Jerusalem (Heb.): Cana.

[48] Dafni A (1984) Edible wild plants of Israel Jerusalem (Heb.): Society for the Protection of Nature in Israel.

[49] Feinbrun-Dothan N (1978) Flora Palaestina. Jerusalem: The Israel Academy of Sciences and Humanities.

[50] Feinbrun-Dothan N (1986) Flora Palaestina. Jerusalem: The Israel Academy of Sciences and Humanities.

[51] Zohary M (1966) Flora Palaestina. Jerusalem: Israel Academy of Sciences and Humanities.

[52] Zohary M (1972) Flora Palaestina. Jerusalem: Israel Academy of Sciences and Humanities.

[53] Kislev ME (1989) Pre-Domesticated Cereals in the Pre-Pottery Neolithic A Period In: Hershkovitz I, editor. People and Culture in Change. Oxford: BAR International Series pp. 147–151.

[54] Snir A, Weiss E (2014) A novel morphometric method for differentiating wild and domesticated barley through intra-rachis measurements. Journal of Archaeological Science 44: 69–75.

[55] Kislev ME, Weiss E, Hartmann A (2004) Impetus for sowing and the beginning of agriculture: Ground collecting of wild cereals. Proceedings of the National Academy of Sciences of the United States of America 101: 2692–2695. 14976246 10.1073/pnas.0308739101 PMC373256

[56] Harlan JR (1967) A wild wheat harvest in Turkey. Archeology 20: 197–201.

[57] Tanno K-i, Willcox G (2012) Distinguishing wild and domestic wheat and barley spikelets from early Holocene sites in the Near East. Vegetation History and Archaeobotany 21: 107–115.

[58] Anderson PC (1999) Experimental cultivation, harvest, and threshing of wild cereals: their relevance for interpreting the use of Epi-Paleolithic and Neolithic artifacts In: Anderson PC, editor. Prehistory of Agriculture. Los Angeles: Institute of Archaeology, University of California pp. 118–144.

[59] Unger-Hamilton R (1999) Experiments in harvesting wild cereals and other plants In: Anderson PC, editor. Prehistory of Agriculture. Los Angeles: Institute of Archaeology, University of California pp. 145–152.

[60] Yamada S (2000) Development of the Neolithic: Lithic Use-wear Analysis of Major Tool Types in the Southern Levant. Cambridge, Massachusetts: Harvard University.

[61] Groman-Yaroslavski I (2014) The Transition to Early Neolithic Economy Reconstructed by Functional Analysis of Blades: A Case Study of Late Natufian and Pre-Pottery Neolithic A Sites in the Salibiya Basin, Southern Jordan Valley. Haifa: University of Haifa.

[62] Kislev ME, Melamed Y, Langsam Y (2006) Plant remains from Tel Batash In: Panitz-Cohen N, Mazar A, editors. Timnah (Tel Batash) III: The Finds from the Second Millennium BCE. Jerusalem: The Hebrew University pp. 295–311.

[63] Zohary D (1969) The progenitors of wheat and barley in relation to domestication and agriculture dispersal in the Old World In: Ucko PJ, Dimbleby GW, editors. The Domestication and Exploitation of Plants and Animals. London: Duckworth pp. 47–66.

[64] Hillman GC, Davies MS (1999) Domestication rates in wild-type wheats and barley under primitive cultivation In: Anderson P, editor. Prehistory of agriculture, new experimental and ethnographic approaches. Los Angeles: The Institute of Archaeology, University of California pp. 70–102.

[65] Kislev ME (2002) Origin of Annual crops by agro-evolution. Israel Journal of Plant Sciences 50: S85–88.

[66] Tanno K-i, Willcox G (2006) How Fast Was Wild Wheat Domesticated? Science 311: 1886 16574859 10.1126/science.1124635

[67] Fuller DQ (2007) Contrasting patterns in crop domestication and domestication rates: recent archaeobotanical insights from the Old World. Annals of Botany 100: 903–924. 17495986 10.1093/aob/mcm048 PMC2759199

[68] Abbo S, Lev-Yadun S, Gopher A (2012) Plant Domestication and Crop Evolution in the Near East: On Events and Processes. Critical Reviews in Plant Sciences 31: 241–257.

