# Supplementary material for: The Origin of Cultivation and Proto-Weeds, Long Before Neolithic Farming
Source: PLoS One. 2015 Jul 22;10(7):e0131422. doi: 10.1371/journal.pone.0131422 (PMC4511808; doi:10.1371/journal.pone.0131422)

**S1 Fig. Seasonal availability of plants and birds found at Ohalo II, based on 68 species of birds [**[**23**](#_ENREF_23)**] and 101 species of plants.**


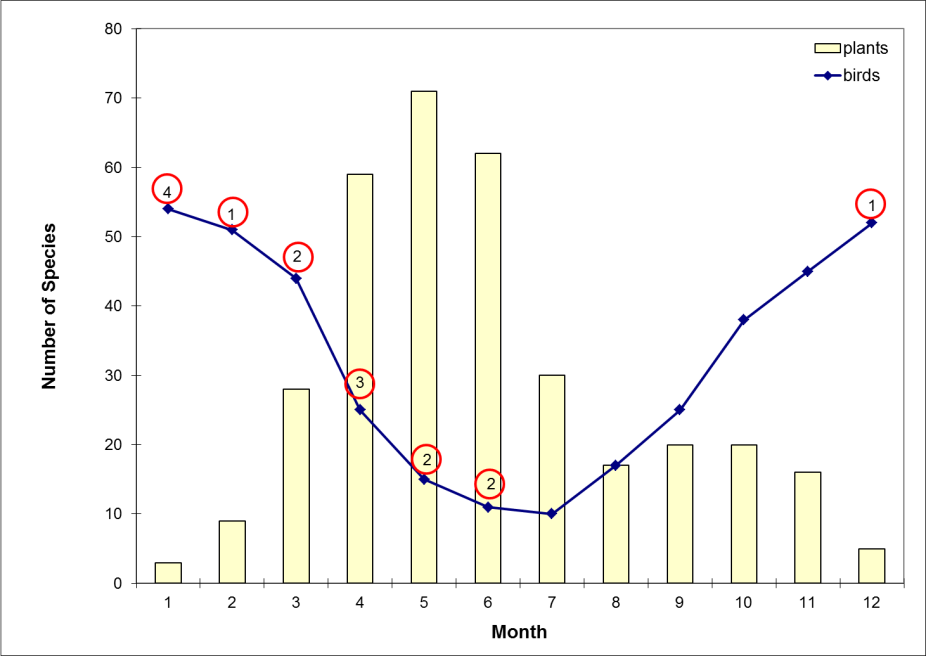

Supplement: S1 Fig — These two types of food resources compensate for each other–birds are more available during autumn/winter while plants prevail in spring/summer. The circled numbers indicate the number of migratory bird species that currently visit the area only once a year. These were eaten from December to June at Ohalo II, and therefore serve as a good indicator of season of occupation of the site. These and other faunal remains point to year–round occupation of Ohalo II. (DOCX) [file pone.0131422.s001.docx]
