# Supplementary figures and images for: The Origin of Cultivation and Proto-Weeds, Long Before Neolithic Farming
Source: PLoS One. 2015 Jul 22;10(7):e0131422. doi: 10.1371/journal.pone.0131422 (PMC4511808; doi:10.1371/journal.pone.0131422)

**S2 Fig. Darnel (*Lolium temulentum*)**.


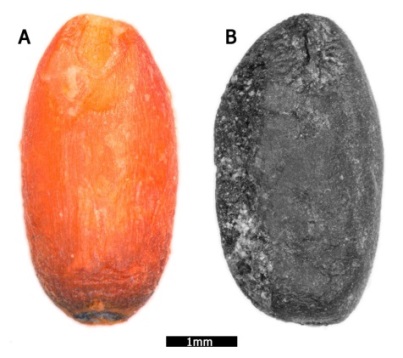

Supplement: S2 Fig — (A) Modern darnel and (B) archaeological darnel from Ohalo II. (DOCX) [file pone.0131422.s002.docx]
